# Dual-specificity tyrosine phosphorylation-regulated kinase 2 (DYRK2) as a novel marker in T1 high-grade and T2 bladder cancer patients receiving neoadjuvant chemotherapy: DYRK2 is associated with survival in bladder cancer

**DOI:** 10.1186/s12894-015-0040-7

**Published:** 2015-06-19

**Authors:** Shunichiro Nomura, Yasutomo Suzuki, Ryo Takahashi, Mika Terasaki, Ryoji Kimata, Yasuhiro Terasaki, Tsutomu Hamasaki, Go Kimura, Akira Shimizu, Yukihiro Kondo

**Affiliations:** Departments of Urology, Nippon Medical School, 1-1-5 Sendagi, Bunkyo-ku, Tokyo 113-8603 Japan; Analytic Human Pathology, Nippon Medical School, 1-1-5 Sendagi, Bunkyo-ku, Tokyo 113-8603 Japan

**Keywords:** DYRK2, Bladder cancer, Prognostic marker, Chemotherapy

## Abstract

**Background:**

To investigate associations between dual-specificity tyrosine phosphorylation-regulated kinase 2 (DYRK2) expression and survival in T1 high-grade or T2 bladder cancer patients treated with neoadjuvant chemotherapy.

**Methods:**

The cohort under investigation comprised 44 patients who underwent neoadjuvant chemotherapy for pT1 high-grade or pT2N0M0 bladder cancer at our institution between 2002 and 2011. Immunohistochemical analysis was used to determine expression of DYRK2 in bladder cancer specimens obtained by transurethral resection before chemotherapy. Relationships between DYRK2 expression and both response to chemotherapy and survival in these patients were analyzed.

**Results:**

DYRK2 expression was positive in 21 of 44 patients (47.7 %) and negative in 23 patients (52.3 %). In total, 20 of 21 DYRK2-positive cases showed complete response to neoadjuvant chemotherapy, whereas 11 of 23 DYRK2-negative cases did not show complete response. Sensitivity and specificity were 62.5 % and 91.7 %, respectively (*P* = 0.0018). In addition, disease-specific survival rate was significantly higher for DYRK2-positive patients than for DYRK2-negative patients (*P* = 0.017). In multivariate analysis, DYRK2 expression level was identified as an independent prognostic factor for disease-specific survival (*P* = 0.029). We also showed that DYRK2 mRNA expression was significantly higher in DYRK2-positive samples by immunohistochemistry than DYRK2-negative samples (*P* = 0.040).

**Conclusions:**

DYRK2 expression level may predict the efficacy of neoadjuvant chemotherapy for T1 high-grade and T2 bladder cancer.

## Background

Radical cystectomy is widely performed to treat muscle-invasive bladder cancer. However, radical cystectomy only results in 5-year survival in about 50 % of patients [1–5]. To improve these unsatisfactory results, the use of peri-operative chemotherapy has been explored. More specifically, the benefits of neoadjuvant chemotherapy have been observed in several trials [6–8].

However, some patients with muscle-invasive bladder cancer do not achieve results even from neoadjuvant chemotherapy. To optimize survival, selecting patients with muscle-invasive bladder cancer who are expected to show a good response to neoadjuvant chemotherapy is important. Various pathological factors have been reported as prognostic markers of poor survival in patients with bladder cancer, but are inadequate for predicting survival in bladder cancer patients. Therefore, molecular markers that better predict survival in T2 bladder cancer patients treated with neoadjuvant chemotherapy are sorely needed.

Dual-specificity tyrosine phosphorylation-regulated kinases (DYRKs) are a subfamily of protein kinases that catalyze their autophosphorylation on tyrosine residues and the phosphorylation of serine/threonine residues on exogenous substrates [9–11]. DYRKs play key roles in the regulation of cell differentiation, proliferation, and survival [12, 13]. Specifically, DYRK2 is associated with cancer survival. DYRK2 phosphorylates p53 at Ser46 during the apoptotic response to DNA damage, thereby promoting cellular apoptosis after genotoxic stress [14]. The presence of DYRK2 may thus predict response to neoadjuvant chemotherapy that induces DNA damage. This finding led us to hypothesize that DYRK2 might be a novel marker of response to neoadjuvant chemotherapy, including cisplatin, at our institution. The present study therefore examined the association between DYRK2 expression and efficacy of neoadjuvant chemotherapy in clinical practice for patients with T1 high-grade or T2 bladder cancer.

## Methods

### Patients and samples

The cohort under investigation comprised 44 patients who underwent neoadjuvant chemotherapy for pT1 high-grade or pT2N0M0 bladder cancer at our institution between April 2003 and February 2011. Having been compiled for research purposes, this group represents patients for whom pretreatment, archival paraffin-embedded tissue blocks and data from complete clinical follow-up were available. Diagnostic work-up included initial transurethral resection of bladder tumor (TURBT), pelvic magnetic resonance imaging (MRI), chest and abdominal computed tomography (CT), and bone scintigraphy. Tumors were graded histologically in accordance with World Health Organization (WHO) classifications and were staged as per the TNM staging system of the Union for International Cancer Control (2009). Histological type was urothelial carcinoma in all cases.

Neoadjuvant intra-arterial chemotherapy was performed after complete TURBT, only after the patient consented to therapy based on our recommendation. Written informed consent was obtained from all patients. Anticancer agents administered as neoadjuvant chemotherapy consisted of cisplatin at 100 mg/m^2^, methotrexate at 30 mg/m^2^, and doxorubicin at 20 mg/m^2^. Our therapeutic protocol comprised two courses of neoadjuvant chemotherapy. Following this, a second TURBT was performed to obtain a biopsy specimen. We assessed the efficacy of neoadjuvant chemotherapy using the pathological results of TURBT. Complete response (CR) was defined as T0 (no evidence of tumor), Ta (noninvasive papillary tumor), or Tis (tumor at a site distant from the original tumor), as in RTOG 99–06 [15]. In cases of CR on the second TURBT, the bladder was preserved, while advanced cases and cases with residual invasive bladder tumors were treated by total cystectomy or systemic chemotherapy [16].

After the second TURBT, cystoscopy and urinary cytological examinations were performed every 3 months for 2 years, every 6 months from 3–5 years, and annually thereafter. Chest radiography and pelvic CT were performed every 6 months for 3 years, and annually thereafter. In cases with visible tumors or hyperemic mucosa in the bladder on cystoscopy or pelvic urinary cytological findings, transurethral biopsy was performed to detect disease recurrence.

This study was carried out in accordance with the Declaration of Helsinki and Good Clinical Practice Guidelines. Approval of the protocol was obtained from the Institutional Review Board of Nippon Medical School, Tokyo, Japan.

### Immunohistochemical analysis

**Fig. 1:**
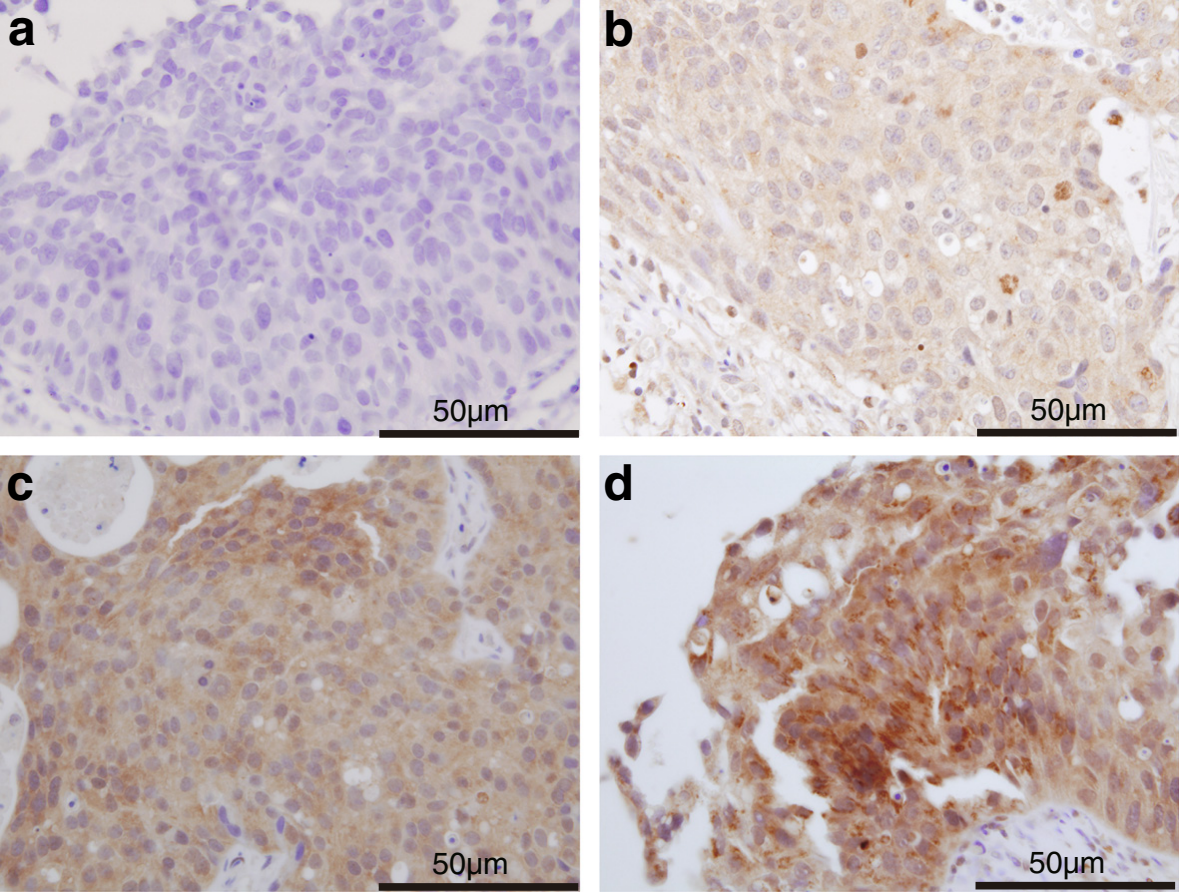
Immunohistochemical staining of paraffin-embedded sections immediately before neoadjuvant chemotherapy with an anti-DYRK2 antibody. DYRK2 protein expression was localized within the cytoplasm. Magnification × 600. **a** DYRK2-negative staining pattern (no staining: score 0). **b** DYRK2-negative staining pattern (weak: score 1). **c** DYRK2-positive staining pattern (moderate: score 2). **d** DYRK2-positive staining pattern (strong: score 3)

DYRK2 expression was determined by immunohistochemical (IHC) staining of paraffin-embedded tissue sections from TURBT specimens immediately before neoadjuvant chemotherapy. The 3-μm-thick sections were deparaffinized, rehydrated using xylene and alcohol, and incubated with 0.3 % H_2_O_2_ to block endogenous peroxidase activity. Before immunostaining, antigen retrieval was performed at 120 °C for 10 min in an autoclave with citrate buffer (pH 6.0). Staining with a polyclonal anti-DYRK2 antibody (AP7534a; dilution, 1:50; Abgent, San Diego, CA, USA) was performed overnight at 4 °C. Histofine Simple Stain Rabbit MAX PO (MULTI; Nichirei, Tokyo, Japan) was used as the secondary antibody in accordance with the manufacturer’s instructions. Color was developed using diaminobenzidine with 0.01 % H_2_O_2_. Hematoxylin was used as a counterstain. Stained tumor tissues were evaluated blindly with respect to clinical patient data. Cytoplasmic staining was considered positive, and staining intensity was scored as 0, 1, 2, or 3, corresponding to no staining, weak, moderate, and strong intensities, respectively (Fig. 1). Percentage scores of cells showing cytoplasmic staining were also counted (0–100 %). Total histochemical score (H-score) was calculated by multiplying the intensity score by the percentage score (0–300). An H-score higher than the median was considered positive. Negative controls were incubated without the primary antibody.

### Analysis of real-time quantitative reverse-transcriptase polymerase chain reaction

Total RNA from formalin-fixed paraffin-embedded tissues was isolated using an Allprep DNA/RNA kit (Qiagen, Tokyo, Japan). The quantity and quality of RNA were evaluated by spectrophotometry. Reverse transcription of RNA to cDNA was achieved using a High-Capacity cDNA Reverse Transcription kit (Applied Biosystems, Foster City, CA, USA). Quantitative gene expression was determined for DYRK2 (Hs00705109_s1) and 18 s (Hs03928990_g1) using gene-specific probes (Applied Biosystems) using TaqMan Fast Advanced Master Mix and the 7900HT Fast Real-time PCR system (Applied Biosystems). PCR conditions were: 5 °C for 2 min and 95 °C for 20 s, followed by 45 cycles at 95 °C for 1 s and 60 °C for 20 s. Data were then quantified using the comparative Ct method for relative gene expression compared with 18S as an endogenous control.

### Statistical analysis

Associations between DYRK2 expression and clinicopathological factors were analyzed using the Fisher’s exact test. Disease-specific survival rates were calculated using the Kaplan–Meier method and differences in survival among groups were compared using log-rank testing. We used Cox proportional hazards regression analysis to assess DYRK2 expression and sex for disease-specific survival. Differences in DYRK2 mRNA between DYRK2-positive and -negative tumors by IHC analysis were determined using the paired *t*-test. *P*-values < 0.05 were considered statistically significant. All statistical analyses were performed using SPSS version 21.0 statistical software (IBM Corp, Armonk, NY, USA).

## Results

### Patient characteristics

Baseline characteristics for all 44 patients are shown in Table 1. The mean age of patients at first TURBT was 70 years (range, 43–84 years) and only eight patients were female. Of the 44 patients, 14 (32 %) showed pT1 high-grade and 30 (68 %) had pT2. Cystectomy was performed after intra-arterial chemotherapy in four patients (9.1 %). With a median follow-up of 47 months, the 5-year survival rate was 82.7 % for all patients. At the time of analysis, 36 patients (81.8 %) were alive and 8 patients (18.2 %) had died of bladder cancer. Overall and disease-specific survival were thus similar.

**Table 1:** Clinicopathological factors of bladder cancer and associations with DYRK2 expression

	Patients (%)	DYRK2 expression		
		Negative	Positive	*P*
All patients	44	23	21	
Age				0.35
<70 years	17 (39 %)	7	11	
≥70 years	27 (61 %)	16	10	
Sex				0.70
Male	36 (82 %)	18	18	
Female	8 (18 %)	5	3	
Pathological T stage				0.27
1	14 (32 %)	5	9	
2a	20 (46 %)	11	9	
2b	10 (23 %)	7	3	
Histological grade				
Low	3 (7 %)	1	2	0.60
High	41 (93 %)	22	19	
Concurrent CIS				0.23
Yes	7 (16 %)	2	5	
No	37 (84 %)	21	16	
Lymphovascular invasion				1.00
Yes	3 (7 %)	2	1	
No	41 (93 %)	21	20	

### Immunohistochemical assessment of DYRK2 expression

DYRK2 was localized in the cytoplasm of bladder tumor cells. Representative cases for the different staining levels (0, 1, 2, and 3) are presented in Fig. 1. Median H-score was 10 (range, 0–230). Therefore, tumors with H-score >10 were deemed DYRK2-positive. Twenty-three specimens (52.3 %) showed low DYRK2 expression, whereas 21 specimens showed high expression (47.7 %).

The relationship between DYRK2 expression and clinicopathological factors is summarized in Table 1. No significant association was observed between DYRK2 expression and the following clinicopathological factors: age, sex, pathological T stage, histological grade, concurrent CIS, or lymphovascular invasion (*P* > 0.05).

### DYRK2 expression and response to Neoadjuvant chemotherapy

Overall, 20 of the 21 DYRK2-positive cases showed CR after neoadjuvant chemotherapy, whereas 11 of the 23 DYRK2-negative cases showed non-CR. The efficacy of neoadjuvant chemotherapy as determined by DYRK2 expression had a sensitivity of 62.5 % and specificity of 91.7 % (*P* = 0.0018, Table 2).

**Table 2:** DYRK2 immunohistochemical staining and clinical response (Fisher’s exact test: *P* = 0.0018)

DYRK2	Clinical response	
Immunoreactive	CR	Non-CR
Positive	20	1
Negative	12	11

*CR* complete response

### DYRK2 expression and survival

**Fig. 2:**
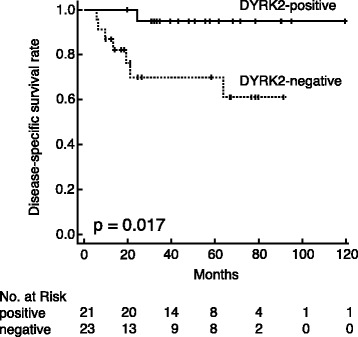
Kaplan–Meier survival analysis in patients positive and negative for DYRK2 expression. Differences in disease-specific survival between subgroups were analyzed by log-rank test. Disease-specific survival was significantly longer for DYRK2-positive patients than for DYRK2-negative patients (*P* = 0.017)

Disease-specific survival was significantly longer for DYRK2-positive patients than for DYRK2-negative patients (*P* = 0.017; Fig. 2). In T2 bladder cancer patients only, DYRK2 expression was associated with increased disease-specific survival (*P* = 0.036). In T1 high-grade bladder cancer patients only, no significant association was observed between DYRK2 expression and disease-specific survival (*P* = 0.157).

Multivariate analysis was performed to evaluate the influence of DYRK2 on disease-specific survival after adjusting for possible confounding factors. From the results shown in Table 1, no clinicopathological factors were significantly correlated with DYRK2. From the results of the univariate analysis, however, DYRK2 and sex significantly correlated with disease-specific survival. Therefore, only DYRK2 and sex were included in the Cox proportional hazards model. DYRK2 expression remained statistically significant (*P* = 0.029), and the hazard ratio (HR) was 11.5 (95 % confidence interval [CI]: 1.29–102; Table 3).

**Table 3:** Univariate and multivariate analysis for disease-specific survival

	Disease-specific survival		
	Univariate analysis	Multivariate analysis	
Characteristics	*P*	HR (95 % CI)	*P*
Age (<70 years vs. ≥70 years)	0.37		
Sex (male vs. female)	0.034	0.15 (0.03-0.79)	0.025
Pathological T stage (T1 vs. T2)	0.18		
Histological grade (low vs. high)	0.38		
Concurrent CIS (Yes vs. No)	0.18		
Lymphovascular invasion (Yes vs. No)	0.35		
DYRK2 expression (positive vs. negative)	0.017	11.5 (1.29-102)	0.029

### DYRK2 mRNA expression in bladder cancer tissue

**Fig. 3:**
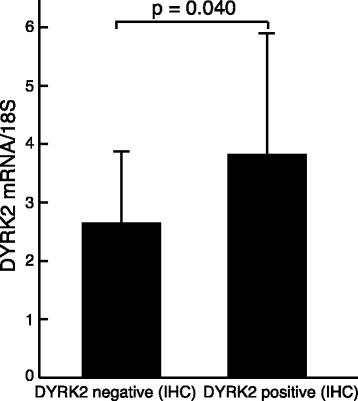
Relative levels of DYRK2 mRNA in DYRK2-positive and -negative cases by IHC analysis. DYRK2 mRNA levels were significantly higher in DYRK2-positive patients than in DYRK2-negative patients (*P* = 0.040)

DYRK2 mRNA expression was assessed in 39 samples. We detected levels of DYRK2 mRNA in DYRK2-positive and DYRK2-negative samples by IHC analysis. Relative mRNA levels of the DYRK2 gene (DYRK2 per 18S, mean ± standard deviation) differed significantly between DYRK2-positive (mRNA 3.82 ± 2.10) and DYRK2-negative patients (mRNA 2.65 ± 1.23). Paired *t*-test showed that mRNA levels were significantly higher in DYRK2-positive patients than in DYRK2-negative patients (*P* = 0.040; Fig. 3).

## Discussion

The present study revealed a significant association between DYRK2 expression and efficacy of neoadjuvant chemotherapy for T1 high-grade and T2 bladder cancer patients. High levels of DYRK2 expression was associated with increased disease-specific survival time in T1 high-grade and T2 bladder cancer patients treated with neoadjuvant chemotherapy. In multivariate analysis, DYRK2 expression levels emerged as independent prognostic markers of survival. DYRK2 may therefore predict prognosis independent of common prognostic factors, such as clinical T stage and histological grade. Moreover, we showed that DYRK2 mRNA expression was significantly higher in DYRK2-positive samples by IHC than DYRK2-negative samples.

DYRK2 is an important factor in cellular apoptosis after genotoxic stress. Taira et al. reported that knock down of DYRK2 function attenuates the apoptosis elicited by DNA damage induced by doxorubicin in vitro [14]. Yamashita et al. reported that DYRK2 may predict progression-free survival in patients with recurrent non-small-cell lung cancer treated using platinum-based chemotherapy [17]. DYRK2 can thus predict response to different chemotherapies, including doxorubicin and cisplatin. As our regimen includes both cisplatin and doxorubicin, DYRK2 may therefore be a useful marker of sensitivity to neoadjuvant chemotherapy at our institution. Furthermore, one of the most popular neoadjuvant chemotherapy regimens includes methotrexate, vinblastine, doxorubicin, and cisplatin (M-VAC), while another popular neoadjuvant chemotherapy regimen consists of gemcitabine and cisplatin (GC). Thus, DYRK2 may predict response to M-VAC and GC. Therefore, trials with M-VAC and GC are needed to confirm this hypothesis.

Few genetic markers have been confirmed to predict survival in bladder cancer patients treated with chemotherapy. Bellmunt et al. reported that excision repair cross-complementing 1 (ERCC1) gene expression may predict survival in patients with bladder cancer treated with platinum-based therapy [18]. Moreover, Font et al. reported that bladder cancer susceptibility 1 (BRCA1) gene expression may predict the efficacy of cisplatin-based neoadjuvant chemotherapy [19]. Finally, Hoffmann et al. reported that high multidrug resistance 1 (MDR1) gene expression was associated with inferior outcomes after cisplatin-based adjuvant chemotherapy for locally advanced bladder cancer [20]. We have also previously reported that Snail expression may predict poor outcomes in bladder cancer patients treated with neoadjuvant chemotherapy [21]. Thus, Use of ERCC1, MDR1, BRCA1, and Snail in combination with DYRK2 may further improve the accuracy of predicting survival in bladder cancer patients treated with neoadjuvant chemotherapy.

We have previously reported that CYFRA 21–1 may be a useful marker for monitoring neoadjuvant chemotherapy [16]. However, this marker cannot predict the efficacy of neoadjuvant chemotherapy prior to administration. The results of the present study indicate that DYRK2 can help identify patients with T1 high-grade and T2 bladder cancer that will respond to neoadjuvant chemotherapy. Therefore, a selective approach using this information could result in patients with high DYRK2 expression receiving neoadjuvant chemotherapy, while those with low DYRK2 expression would undergo radical cystectomy. Prospective studies applying this approach are needed in the future.

In T2 bladder cancer patients only, DYRK2 expression was associated with increased disease-specific survival time (*P* = 0.036). However, no significant association was observed between DYRK2 expression and disease-specific survival in only T1 high-grade bladder cancer patients (*P* = 0.157). However, a trend toward longer disease-specific survival was observed. Further studies with a large cohort of T1 high-grade bladder cancer patients are warranted to confirm this result.

One limitation of the present study is that we have not shown a direct role of DYRK2 in bladder cancer. However, our DYRK2 staining results with clinical samples suggest that the abundance of DYRK2 is associated with the response to neoadjuvant chemotherapy. Other limitations of this study include that the sample size for DYRK2 immunohistochemical analysis was very small, the study was retrospective, and our regimen does not represent standard chemotherapy. More detailed studies are needed to address these limitations.

## Conclusions

Although the sample size of this study was small, our results indicate that DYRK2 might represent a new molecular marker for predicting the efficacy of neoadjuvant chemotherapy in T1 high-grade and T2 bladder cancer. Further careful study is needed to confirm our preliminary results.

## Abbreviations

CI: Confidence interval
RTOG: Radiation Therapy Oncology Group
CIS: Carcinoma *in situ*
